# TTF-1 regulates α_5 _nicotinic acetylcholine receptor (nAChR) subunits in proximal and distal lung epithelium

**DOI:** 10.1186/1465-9921-11-175

**Published:** 2010-12-09

**Authors:** Paul R Reynolds, Camille H Allison, Charles P Willnauer

**Affiliations:** 1 Department of Physiology and Developmental Biology, Brigham Young University, Provo, UT 84602, USA

## Abstract

**Background:**

Nicotinic acetylcholine receptors (nAChRs) are ligand-gated ion channels comprised of five similar subunits that influence signal transduction and cell turnover. α_5 _is a structural subunit detected in many non-neuronal tissues; however, its function during pulmonary development is unknown.

**Results:**

α_5 _was assessed by immunohistochemistry and RT-PCR in mouse lungs from embryonic day (E)13.5 to post-natal day (PN)20. From E13.5 to E18.5, α_5 _expression was primarily observed in primitive airway epithelial cells while mesenchymal expression was faint and sporadic. α_5 _expression was detected throughout the proximal lung at PN1 and extensively expressed in the peripheral lung at PN4, an early stage of murine alveologenesis. An interesting shift occurred wherein α_5 _expression was almost undetectable in the proximal lung from PN4-PN10, but significant localization was again observed at PN20. Transcriptional control of α_5 _was determined by assessing the activity of reporters containing 2.0-kb and 850-bp of the mouse α_5 _promoter. Because perinatal expression of α_5 _was abundant in bronchiolar and alveolar epithelium, we assessed transcriptional control of α_5 _in Beas2B cells, a human bronchiolar epithelial cell line, and A-549 cells, an alveolar type II cell-like human epithelial cell line. Thyroid Transcription Factor-1 (TTF-1), a key transcription regulator of pulmonary morphogenesis, significantly increased α_5 _transcription by acting on both the 2.0-kb and 850-bp α_5 _promoters. Site-directed mutagenesis revealed that TTF-1 activated α_5 _transcription by binding specific TTF-1 response elements. Exogenous TTF-1 also significantly induced α_5 _transcription.

**Conclusions:**

These data demonstrate that α_5 _is specifically controlled in a temporal and spatial manner during pulmonary morphogenesis. Ongoing research may demonstrate that precise regulation of α_5 _is important during normal organogenesis and misexpression correlates with tobacco related lung disease.

## Background

Mechanisms that control pulmonary development involve highly coordinated processes that require precise reciprocal interactions between endodermally derived respiratory epithelium and the surrounding splanchnic mesenchyme. These interactions are predominantly mediated by cell surface receptors and specific ligands elaborated by communicating cells of both germinal origins. Initial primordial lung buds undergo branching to form the main bronchi and extensive subsequent branching events lead to the formation of the intrapulmonary conducting and peripheral lung airways. Distinct populations of differentiated respiratory epithelial cell types then arise, producing a morphologically dynamic arrangement of cells that in due course influence pulmonary function and respiratory efficiency. The temporal and spatial pattern of cell surface receptor expression must therefore be specifically controlled in order to orchestrate mechanisms of proliferation, migration, and differentiation essential during lung morphogenesis.

Thyroid transcription factor (TTF)-1 is a member of the homeodomain-containing Nkx2 family of transcription factors. TTF-1 is expressed in the lung, thyroid, ventral forebrain, and the pituitary [1-3]. While TTF-1 mRNA is initially detected in the mouse at E10 [4] its pattern of expression principally localizes to the lung periphery during pulmonary development [2]. TTF-1 activates the expression of genes critical to lung development and function such as surfactant proteins (SPs), Clara cell secretory protein (CCSP), various growth factors, and molecules required for normal host defense and vasculogenesis [4,5]. Inactivation of TTF-1 causes tracheo-esophageal fistulae and impairment of pulmonary branching, leading to severe lung hypoplasia [6]. In concert with other transcription factors, TTF-1 binds TTF-1 response elements (TREs) in promoters of target genes in order to regulate gene expression and cell differentiation during lung morphogenesis. While our preliminary studies and the work of others reveal that α_5 _is detected in cells known to express TTF-1 [7-9], no regulatory mechanism has been proposed linking the two in the lung to date.

Neuronal and non-neuronal nicotinic acetylcholine receptors (nAChRs) combine with glycine, GABA_A_, and 5HT3 receptors to form a family of ligand-gated ion channels [10]. nAChRs are pentameric oligomers composed of five related subunits arranged around a central ion channel that allows flow of calcium or sodium following ligand binding. Subsequent to ligand interaction, pathways associated with intracellular signal transduction, proliferation, and apoptosis are induced [11-13]. Several receptor subunits have been identified and are classified as either agonist binding (α_2_, α_3_, α_4_, α_6_, α_7_, α_9 _and α_10_) or structural (α_5_, β_2_, β_3 _and β_4_) [14,15]. Work performed previously by our laboratory demonstrated that α_7 _nAChRs, homomeric receptors composed of five α_7 _subunits, are temporally controlled in the lung during development and are transcriptionally regulated by TTF-1 [16].

In the current investigation, we report that α_5 _nAChR subunits are expressed in subsets of pulmonary epithelial cells during stages of lung morphogenesis and that these receptor subunits are regulated by TTF-1. This research adds additional insight into TTF-1 regulation of subunits involved in nAChR assembly by joining α_5 _and α_7 _in conserved regulatory pathways. Furthermore, because comparisons between the human α_5 _gene and the α_5 _gene in several other species reveal remarkable conservation, TTF-1 and its homologues may be common transcriptional regulators involved in controlling the precision of α_5 _nAChR expression in the lung.

## Methods

### Mouse Models

α_5 _expression was assessed from E13.5 to PN20 in lungs from wild type and TTF-1 null mice, each in a C57Bl/6 background. Dr. Jeffrey Whitsett at the Cincinnati Children's Hospital Medical Center (CCHMC) generously gifted TTF-1 null mice. Animal husbandry and use followed protocols approved by the Institutional Animal Care and Use Committee at CCHMC and Brigham Young University.

### Antibodies

A rabbit α_5 _polyclonal antibody (generated and kindly gifted by Scott Rogers and Lorise Gahring at the University of Utah) was generated against epitopes in the cytoplasmic domain of the α_5 _protein and has been demonstrated to interact with tissues embedded in paraffin [17,18]. Antibody specificity was confirmed using immunoblotting and ELISA, revealing that the antiserum reacts only with the α_5 _subunit protein to which it was made [19]. While data revealing positive immunostaining for a subset of nAChR subunits in brain samples from both wild type and subunit null animals exists [20,21], there are no published reports demonstrating such effects in lung tissue or employing α_5 _specific antibodies. The α_5 _IgG was used at a dilution of 1:800. A rabbit polyclonal antibody raised against Clara Cell Secretory Protein (CCSP) generated at the CCHMC was used at a dilution of 1:1600 to identify Clara cells in the conducting airways. A rabbit polyclonal antibody for TTF-1 was also generated at CCHMC and used to localize type II alveolar epithelial cells (ATII) at a dilution of 1:1000. Specificity of the CCSP and TTF-1 antibodies was determined by Western blot analysis (not shown).

### Immunohistochemistry

Immunohistochemical staining for α_5_, CCSP, and TTF-1 were performed using standard techniques [22,23]. Briefly, 5-μm paraffin sections from six mice per group were deparaffinized and rehydrated. Sections were treated with 3% hydrogen peroxide in methanol for 15 min to quench endogenous peroxidase. Development in NiDAB was followed by incubation in Tris-cobalt, which enhanced antigen localization, and by counterstaining with nuclear fast red. Sections were then dehydrated in a series of ethanols, washed in three changes of xylene, and mounted under coverslips with Permount. Control sections were incubated in blocking serum alone.

### Plasmid Construction and Mutagenesis

Primers were designed to retrieve 2.0-kb or 0.85-kp of the mouse α_5 _promoter by polymerase chain reaction (PCR) using the Expand High Fidelity PCR System (Roche, Indianapolis, IN). The amplified α_5 _promoter fragment was directionally cloned into the pGL4.10-basic luciferase reporter plasmid (Promega, Madison, WI) and verified by sequencing. Site-directed mutagenesis of potential TTF-1 binding sites was performed by using the reporter construct (pGL4.10-0.85-kb α_5_) and the QuickChange™ Site-Directed Mutagenesis kit (Stratagene, La Jolla, CA). Briefly, synthetic oligonucleotides containing the desired mutation for TTF-1 (CAAT→GGGG) were extended during PCR amplification. The products were digested with *Dpn*I to remove the wild-type DNA. The nicked vector DNA was then transformed into XL1-blue supercompetent cells and repaired. All constructs were verified by nucleotide sequencing.

### Transfection and Reporter Gene Assays

Functional assays of reporter gene constructs were performed by transient transfection of Beas2B and A-549 cells using FuGENE-6 reagent (Roche). Beas2B is a transformed human bronchiolar epithelial cell line and A-549 is a human pulmonary adenocarcinoma cell line characteristic of ATII cells. Cells in 35-mm dishes at 40-50% confluence were transfected with four plasmids at the following concentrations: 300 ng pRSV-βgal, 100 ng pGL4.10-2.0-kb α_5 _or pGL4.10-0.85-kb α_5_, 100-400 ng pCMV-TTF-1 and pCDNA control vector to bring total DNA concentration to 1.2 μg. The cells were allowed to grow to confluence (48 hr), washed with cold PBS, lysed, and snap frozen for several hours. The plates were scraped and centrifuged, and the cleared supernatant was used for both β-gal and luciferase assays. Reporter assays were normalized for transfection efficiency based on the β-gal activity [22]. Luciferase activity was determined in 10 μl of extract at room temperature with 100 μl of luciferase substrates A and B (BD Biosciences, San Jose, CA) for 10 sec after a 2-sec delay in a Moonlight™ 3010 luminometer (BD Biosciences).

### RT-PCR

In order to assess α_5 _mRNA expression throughout development, total RNA was isolated from whole mouse lungs at various time points with the Absolutely RNA^® ^RT-PCR Miniprep Kit (Stratagene) and DNase treated. Because α_5 _was immunolocalized in bronchioles and alveoli, induction of α_5 _mRNA expression was similarly assessed in Beas2B and A-549 cells following transfection with 400 ng pCMV-TTF-1 or control pCDNA vector. 2-μg of total RNA was reverse transcribed using the SuperScript^® ^III First-Strand Kit according to the manufacturer's instructions (Invitrogen). PCR was performed with 2-μl aliquots of the generated cDNA using Taq polymerase (Roche, Indianapolis, IN) and experiments included no template (lacking cDNA) and no RT (without reverse transcriptase) controls (not shown). Products were electrophoresed on a 1.5% agarose gel with appropriate molecular weight standards. Bands were quantified using Un-Scan-it™ gel digitizing software (Silk Scientific, Orem, UT). Gene expression was assessed in three replicate pools and representative data is shown. Primers used for the PCR reactions include α_5 _forward (5'-CTT CAC ACG CTT CCC AAA CT-3') and reverse (5'-CTT CAA CAA CCT CAC GGA CA-3') and GAPDH forward (5'-CGT CTT CAC CAC CAT GGA GA-3') and reverse (5'-CGG CCA TCA CGC CAC AGC TT-3'). PCR parameters included an initial heating at 94°C for 5 m. α_5 _and GAPDH were amplified via 30 cycles at 94°C for 45 s, 57°C for 45 s, and 72°C for 45 s. All amplifications were followed by a 7-min extension at 72°C.

### Statistical Analysis

Results are presented as the means ± S.D. of six replicate pools per group. Means were assessed by one and two-way analysis of variance (ANOVA). When ANOVA indicated significant differences, student t tests were used with Bonferroni correction for multiple comparisons. Results are representative and those with p values < 0.05 were considered significant.

## Results

### α_5 _nAChR Expression During Lung Development

**Figure 1:**
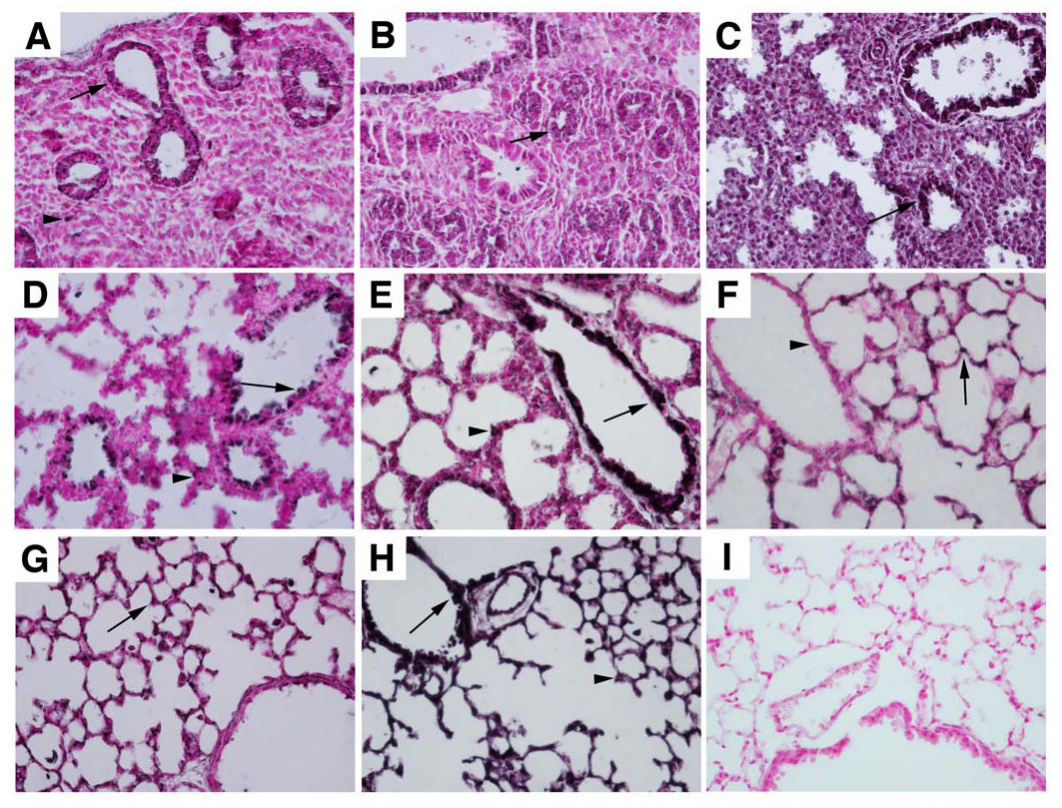
**Immunostaining revealed distinct patterns of α_5 _expression in the lung during organogenesis**. A. α_5 _was expressed at E13.5 in primitive airway epithelium (arrow) and sporadically in pulmonary mesenchyme (arrowhead). B and C. During the late pseudoglandular period of development (B, E15.5) and early saccular stage of development (C, E17.5), α_5 _continued to localize to pulmonary epithelium (arrows). D. At E18.5, α_5 _expression was detected in larger airways (arrows) as well as in primitive respiratory epithelium (arrowhead). E. At PN1, α_5 _expression was markedly detected in proximal lung airways (arrow) and only minimally detected in the peripheral lung (arrowhead). F. At the commencement of alveologenesis (PN4), α_5 _localized to lung parenchyma (arrow) and was noticeably absent in the airways (arrowhead). G. At PN10, α_5 _expression persisted in the respiratory compartment (arrow). H. After alveologenesis progressed to PN20, α_5 _was abundantly expressed throughout the lung, being detected in proximal (arrow) as well as distal pulmonary epithelium (arrowhead). I. PN20 sections incubated without primary α_5 _antibody revealed no immunoreactivity. Six mice were included in each group and representative images at 40× magnification are shown.

**Figure 2:**
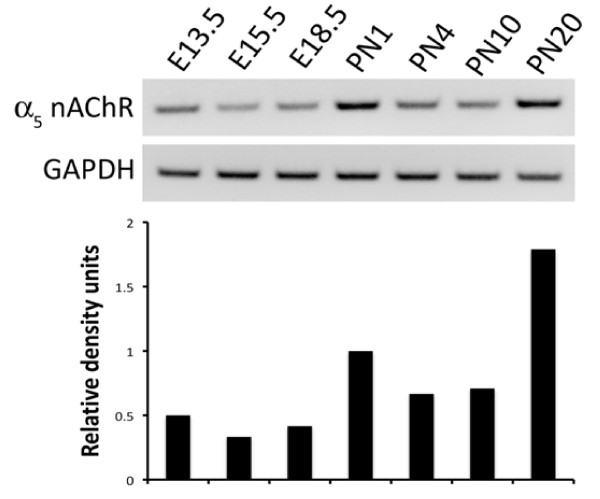
**α_5 _mRNA expression in the lung during organogenesis**. Semi-quantitative RT-PCR analysis revealed variable abundance of α_5 _mRNA expression in whole mouse lung from E13.5 to PN20. Band densities were assessed and normalized after standardization of GAPDH band densities to 1. Although samples were screened from 6 mice at each time point, only a representative single band is shown.

The distribution of α_5 _was assessed in mouse lung by immunohistochemistry from E13.5 to PN20. At E13.5 (Figure 1A) and E15.5 (Figure 1B), α_5 _was predominantly observed in primitive airway epithelial cells and sporadically detected in pulmonary mesenchyme. While mesenchymal staining diminished through E18.5, α_5 _expression in pulmonary epithelium increased and was restricted to luminal cell surfaces (Figure 1D). α_5 _expression was abundantly detected in proximal lung epithelial cells at PN1 (Figure 1E), however, by PN4, α_5 _expression was only detected in the peripheral respiratory region of the lung (Figure 1F). Staining at PN4, a period that coincides with the onset of alveologenesis, revealed α_5 _expression in cells located near alveolar septa characteristic of ATII localization. This pattern of expression in respiratory epithelial cells and minimal to no staining in the proximal lung persisted through PN10 (Figure 1G). BY PN20, α_5 _expression was detected throughout the lung, with abundant immunolocalization in proximal airway epithelium as well as in the respiratory compartment (Figure 1H). No staining was observed in sections stained without primary antibody (Figure 1I). The patterns of α_5 _expression obtained by immunostaining corresponded with α_5 _mRNA expression from E13.5 to PN20 as revealed by semi-quantitative RT-PCR analysis (Figure 2).

**Figure 3:**
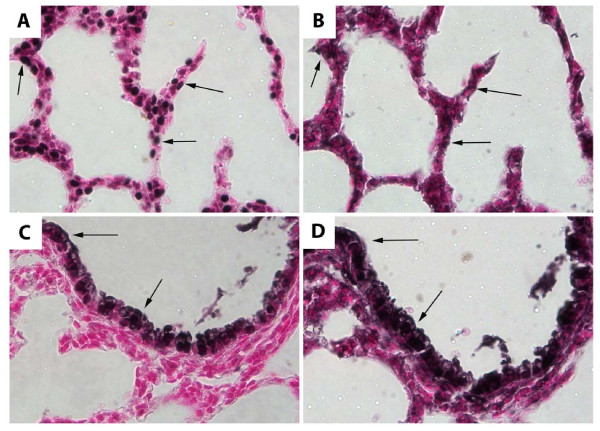
**α_5 _was expressed by and ATII cells Clara cells at PN1**. Staining for TTF-1 (A) and α_5 _(B) in serial sections from PN1 mouse lung revealed α_5 _is co-expressed with TTF-1 in many ATII cells, but not all (arrows). Staining for CCSP (C) and α_5 _(D) also identified consistent α_5 _expression in bronchiolar epithelium at PN1 (arrows). All images are at 80× original magnification.

To identify epithelial cells that express α_5_, immunohistochemistry was performed on serial sections at PN1. Staining serial sections with TTF-1 (Figure 3A), an ATII cell marker, and α_5 _(Figure 3B), revealed α_5 _expression in ATII cells with nuclear staining for TTF-1. While α_5 _was expressed in many ATII cells (Figure 3A and 3B, arrows) not all ATII cells were identified with α_5 _staining. Localization in serial sections was also performed with CCSP, a Clara cell specific marker that identifies non-ciliated Clara cells in the proximal lung that slightly protrude into the airway lumen (Figure 3C). α_5 _staining (Figure 3D) appeared to be associated with many CCSP-secreting Clara cells in pulmonary bronchioles (Figure 3C).

### TTF-1 Regulates α_5 _Transcription In Vitro

**Figure 4:**
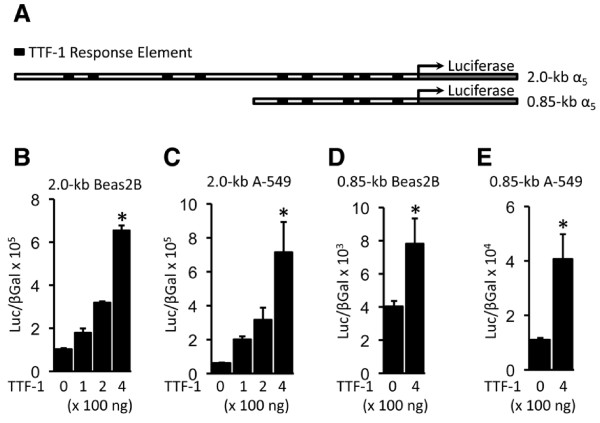
**TTF-1 activated α_5 _transcription in bronchiolar and alveolar epithelial cell types**. A. Schematic of α_5 _luciferase reporters containing 2.0-kb or 0.85-kb mouse α_5 _promoter sequences that include putative TTF-1 response elements (TREs, black rectangles). B and C. TTF-1 dose-dependently induced α_5 _transcription by acting on a 2.0-kb α_5 _reporter in Beas2B (B) and A-549 (C) cells. D and E. TTF-1 also induced significant increases in α_5 _transcription via interaction with a truncated 0.85-kb α_5 _reporter. Significant differences in luciferase levels compared to reporter alone are noted at P ≤ 0.05 (*).

Because there were interesting shifts in the expression of α_5 _by ATII cells at various developmental time points (Figure 1E,F,G,H), experiments were planned that tested whether TTF-1 transcriptionally regulates α_5 _expression. An assessment of the mouse α_5 _promoter sequence revealed the locations of nine potential TTF-1 regulatory elements (TREs) in the 2.0-kb promoter fragment and five in the 0.85-kb fragment (Figure 4A). Because α_5 _experienced profound expression changes from proximal lung (Figure 1E) to distal lung (Figure 1F and 1G) before returning to the proximal lung (Figure 1H), we tested the degree of TTF-1 regulation in both bronchiolar epithelium (Beas2B) and ATII-like alveolar epithelial cells (A-549). TTF-1 (100-400 ng) activated the 2.0-kb α_5 _promoter in a dose-dependent manner in both Beas2B and A549 cells (Figure 4B and 4C). TTF-1 also significantly induced transcription of α_5 _in both cell types when a truncated reporter that contains only 0.85-kb of the promoter was transfected (Figure 4D and 4E).

**Figure 5:**
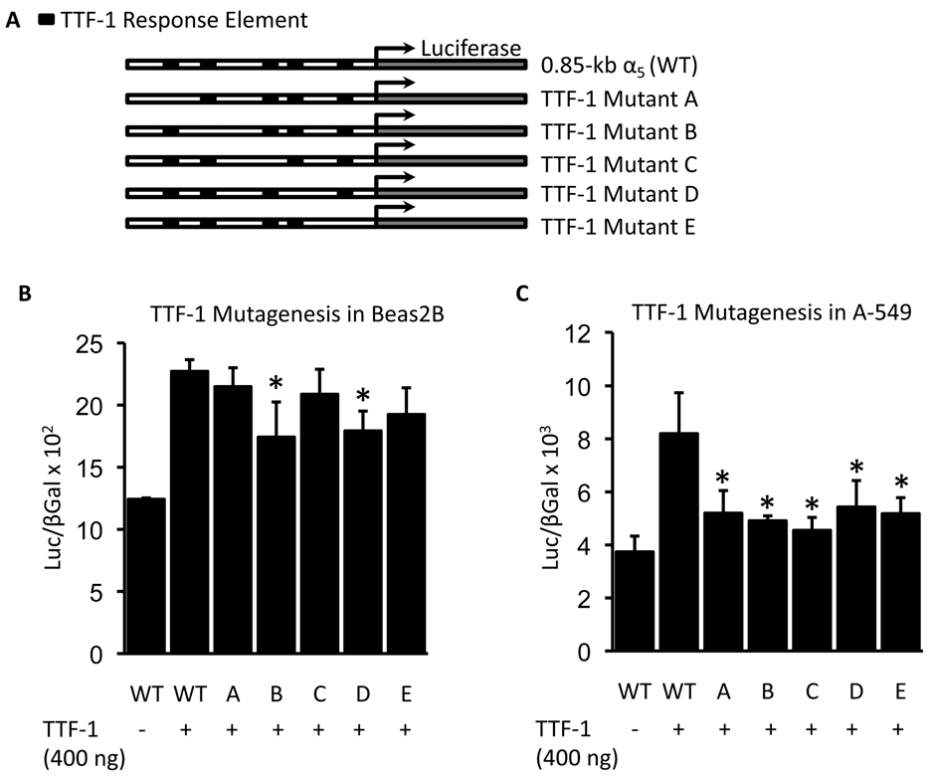
**TTF-1 induced α_5 _transcription via interaction with putative TTF-1 response elements (TREs)**. A. Schematic of a wild type (WT) α_5 _luciferase reporter containing the 0.85-kb mouse α_5 _promoter sequence and reporters that contain a 0.85-kb α_5 _promoter with each sequential TRE targeted by site-directed mutagenesis (Mutants A-E). B. TTF-1-mediated increases in α_5 _transcription were significantly diminished in Beas2B cells when the second or fourth TREs were mutated. C. Each TRE was demonstrated to be significant in regulating TTF-1-mediated α_5 _transcription in ATII-like A-549 cells. Significant decreases in TTF-1 induced luciferase activity resulting from each mutant reporter compared to WT + TTF-1 are noted at P ≤ 0.05 (*).

To identify potential TREs that are critical in TTF-1-mediated control of α_5 _transcription, site-directed mutational analysis was performed resulting in the ablation of putative TREs (Figure 5A). In Beas2B cells, mutation of the second and fourth TRE resulted in a significant decrease in TTF-1-induced transcription compared to the wild type promoter transfected with TTF-1 (Figure 5B). Experiments involving A-549 cells revealed that mutation of any of the five TREs resulted in a significant decrease in TTF-1-induced transcription (Figure 5B).

### TTF-1 Mediates α_5 _Expression In Vitro

**Figure 6:**
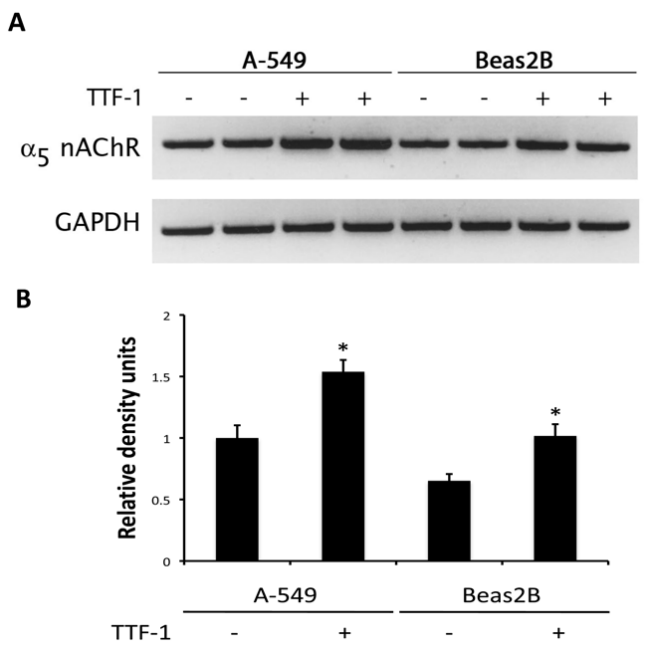
**α_5 _mRNA expression was induced by TTF-1 in ATII-like A-549 cells and bronchiolar epithelium-like Beas2B cells**. A. By semi-quantitative RT-PCR analysis, A-549 and Beas2B cells endogenously express α_5_. Transfection with a TTF-1 expression vector 24 hours prior to mRNA isolation, reverse transcription, and PCR amplification resulted in detectible increases in α_5 _expression. Representative examples are shown. B. Band densities from six replicates per group were assessed and normalized after standardizing GAPDH band density to 1. When all six replicates were assessed, a significant difference in α_5 _expression was detected between TTF-1 and mock transfected cells (*P ≤ 0.05).

In order to further assess the effects of TTF-1 on α_5 _expression, Beas2B and A-549 cells were transfected with TTF-1 and α_5 _was assessed by RT-PCR. In the absence of exogenous TTF-1, Beas2B and A-549 cells both expressed detectible levels of α_5 _(Figure 6). Transfection of TTF-1 24 hours before RNA isolation induced a significant increase in α_5 _mRNA expression in both A-549 and Beas2B cells (Figure 6).

### TTF-1 Targeting Impairs α_5 _Expression In Vivo

**Figure 7:**
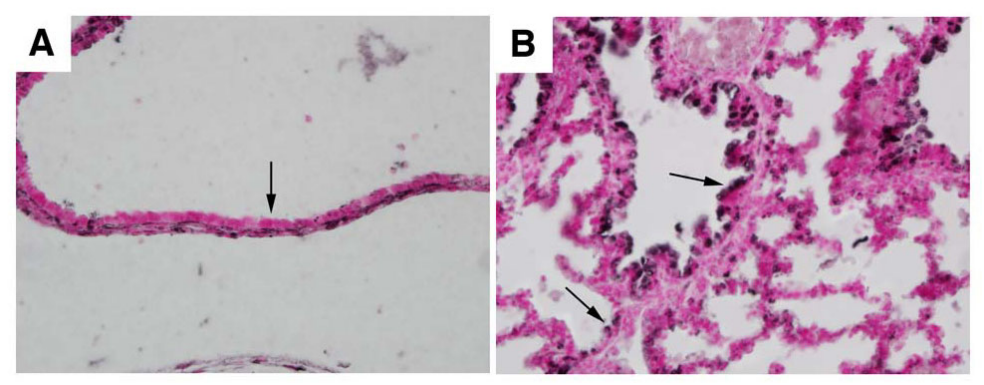
**α_5 _expression was significantly reduced in pulmonary epithelium from E18.5 TTF-1 null mice compared to age-matched with type controls**. A. α_5 _immunostaining in TTF-null mouse lung revealed almost complete ablation of α_5 _expression in pulmonary epithelium (arrow). B. Staining for α_5 _demonstrated marked expression in proximal and distal pulmonary epithelium (arrows). All images are at 40× original magnification.

Because our data demonstrate that α_5 _is expressed in pulmonary epithelium at E18.5 (Figure 1D) and its expression is regulated by TTF-1 (Figures 4, 5, 6), we determined α_5 _expression in TTF-1 null mice. TTF-1 null mice die at birth due to significantly reduced branching morphogenesis and severe lung hypoplasia. Expression of α_5 _in pulmonary epithelium in the lungs of TTF-1 null mice (Figure 7A, arrow) was nearly undetectable when compared to intense α_5 _localization observed in age-matched wild type control lung samples (Figure 7B, arrows).

## Discussion and Conclusions

The temporal-spatial distribution of α_5, _a member of the nicotinic acetylcholine receptor subunit family, was determined during embryonic and postnatal lung development. Various epithelial cell populations expressed α_5 _protein in both the conducting and peripheral air spaces. α_5 _was primarily expressed in respiratory epithelial cells during the embryonic, pseudoglandular, cannalicular, and saccular stages of lung development. In addition to expression in the peripheral lung, α_5 _was also detected perinatally in distinct populations of bronchiolar epithelial cells. Conducting airway epithelial cell expression persisted throughout lung morphogenesis except from PN4 to PN10, a period that coincides with significant parenchymal differentiation in the alveolar stage of lung development. Immunolabeling of α_5 _in the fetal lung was observed primarily on luminal epithelial cell membranes suggesting that α_5 _accumulates on apical cell surfaces in order assemble receptors needed in the postnatal lung. Alternatively, apical expression may suggest that α_5 _subunits combine *in utero *to form functional nAChRs which bind ligand and signal events that are essential during organogenesis. Several groups have shown that nAChRs are expressed in airway epithelium and that they form functional receptors as demonstrated by electrophysiological analyses [8,24,25]. Localization of α_5 _with cells that express CCSP and TTF-1 suggests that α_5 _is regulated by TTF-1 and, therefore, may play a role in the mediation of paracrine signaling between respiratory epithelial cells during pulmonary morphogenesis.

Intriguing aspects of functional pulmonary nAChRs *in utero *are data related to acetylcholine (ACh) as a local signaling molecule synthesized by many non-neuronal cells [26]. In order for ACh to function as a signal in the lung, ACh must be synthesized and secreted locally. Choline is incorporated into pulmonary bronchiolar cells by a choline high-affinity transporter (CHT), synthesized into ACh by choline acetyl transferase (ChAT), and packaged into transport vesicles by a vesicular ACh transporter (VAChT) [26]. Availability of choline in the lung is also possible due to its derivation during the recycling of surfactant proteins and membranes [27]. In addition to acetylation during the generation of ACh, choline has also been demonstrated to be an agonist for a subset of ligand binding nAChR subunits such as α_7 _[28]. While evidence for choline and acetylcholine ligation primarily identifies with the biology of α_7_, the possibility exists that similar agonists interact with receptors structurally maintained by α_5_.

α_5 _was co-expressed with TTF-1 in epithelial cells that contribute to primordial tubules early in lung development [29]. TTF-1 regulates cytodifferentiation and formation of functional respiratory epithelium [5]. Several additional co-expressed transcriptional regulators such as GATA-6 and FoxA2 are also observed in airway epithelium during the period from E13.5 to 15.5 [3,30]. Recent preliminary studies performed in our laboratory reveal that GATA-6 and FoxA2, both transcriptional targets of TTF-1, also individually and synergistically activate the α_5 _promoter, suggesting complex interplay between TTF-1 and other important transcription factors. TTF-1 and various co-regulators such as GATA-6 and FoxA2 interact during the regulation of specific genes critical to lung function, including CCSP, surfactant proteins, growth factors, and VEGFa and VEGFr2 essential in vasculogenesis [31]. While additional research is still necessary, the observations that α_5 _was transcriptionally induced by TTF-1 via interaction with specific promoter response elements and significantly diminished in TTF-1 null mouse lung reveals the importance of TTF-1 in the orchestration of α_5 _regulation. This research also demonstrates that α_5 _and other nAChR subunits such as α_7 _[16] may contribute to an expanding group of important developmental genes regulated by TTF-1. Furthermore, because the α_5 _gene and message maintain remarkable conservation across species (Table 1), TTF-1 and its homologues may influence common transcriptional mechanisms involved in the defined temporal and spatial pattern of α_5 _nAChR expression in the lung.

**Table 1:** Percent homology between human α5 nAChR subunits and other species

Species	Protein%	DNA%
*Homo sapiens*		
vs. *Pan troglodytes *(chimpanzee)	98.9	99.4
vs. *Canis lupus *familiaris (domestic dog)	91.4	83.3
vs. *Bos Taurus *(cow)	90.5	89.6
vs. *Mus musculus *(mouse)	90.7	84.3
vs. *Rattus norvegicus *(rat)	89.6	83.3
vs. *Gallus gallus *(chicken)	85.6	79.5
vs. *Danio rerio *(zebrafich)	77.1	69.3

Even though TTF-1 specifically induced significant α_5 _expression in pulmonary epithelium, the temporal-spatial distribution of TTF-1 and α_5 _during lung development were not completely identical. For example, whereas TTF-1 is an epithelium-specific transcription factor, α_5 _protein was expressed in both the epithelium and mesenchyme at E13.5. The expression of α_5 _is therefore likely regulated by the activity of several transcription factors with overlapping expression patterns. Because TTF-1 regulates target gene expression in concert with other regulatory factors including GATA-6, FoxA2, NF-1, RAR, and AP-1 [31], it is likely that the temporal-spatial distribution of α_5 _expression is influenced in a complex manner by a host of transcription factors.

Nicotinic cholinergic signaling via α_5 _nAChR subunits in airway epithelial cells is likely affected by nicotine. Published reports demonstrate that cells exposed to environmental tobacco smoke, or equal concentrations of nicotine, induce sequential severalfold increases in α_5 _and α_7 _expression [32]. Plasma nicotine levels in smokers fluctuate between 10 and 200 nM and epithelial cells directly exposed to smoke may experience nicotine levels that are 5-10-fold greater [33,34]. Exposure to cigarette smoke during pregnancy adversely affects lung development as manifested by significantly reduced branching morphogenesis [35], increased respiratory illness [36], altered pulmonary function [37], and permanent airway obstruction in the proximal lung [38]. Nicotine crosses the placenta and directly affects lung development *in utero *via interaction with nAChRs in the developing and post-natal lung. Our studies demonstrate that while receptors that contain α_5 _are expressed in populations of epithelial cells during lung development, receptor availability may contribute to adverse lung development and morphological perturbation when noxious ligands are present.

Recently the α_5 _gene (CHRNA5) and other receptor subunits located in the chromosome 15q24-25 region have been the topics of intense investigation due to a correlation between an α_5 _variant and nicotine dependence [39]. While research is ongoing, analysis of this specific chromosomal locus reveals that α_5 _and its variants significantly influence susceptibility to smoke related lung cancer and chronic obstructive pulmonary disease (COPD) [39-41]. Understanding the developmental role of α_5 _and TTF-1-mediated mechanisms that control its precise pattern of expression during lung organogenesis will prove beneficial in elucidating the role of α_5 _in the progression of lung disease commenced *in utero *by tobacco exposure.

In conclusion, α_5 _nAChR subunits are expressed in specific epithelial cell types in the lung during development. α_5 _expression is developmentally regulated by several factors including TTF-1, a molecule centrally involved in normal lung formation. Our data reveals specific regulation of α_5 _expression by TTF-1; however, such expression may be altered by nicotine exposure. While nicotine may directly influence normal cholinergic signaling during morphogenesis that involves α_5_-containing nAChRs, the misregulation of α_5 _may also predispose individuals to lung cancer and COPD.

## Competing interests

The authors declare that they have no competing interests.

## Authors' contributions

CHA generated plasmids and assisted with *in vitro *reporter gene assays. CPW performed immunohistochemistry, reporter gene assays, RT-PCR analysis and assisted in manuscript preparation. PRR conceived of the study and supervised in its implementation, interpretation, and writing. All authors approved of the final manuscript.
